# Market-based tools for cattle sustainability

**DOI:** 10.1093/af/vfab044

**Published:** 2021-09-06


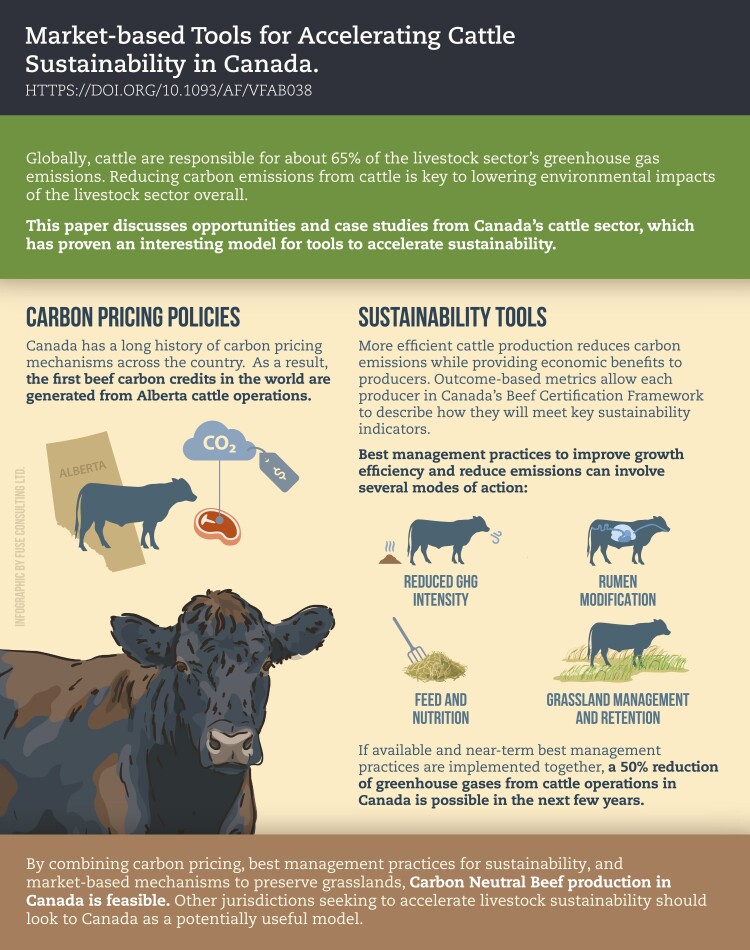


Sustainability in livestock production systems will play a critical role in reaching the Sustainable Development Goals developed by the United Nations (Varijakshapanicker et al., 2019). Globally, progress towards sustainable livestock production has and will continue to focus on environmental impacts, livestock breeding and management practices, education, and policy and market changes that support and incentivize these production systems (Johnson, 2021; Haugen-Kozyra, 2021). Moving toward carbon-neutral livestock production will require on-going, collaborative work between producers, scientists, policymakers, and retailers.
